# Current Research of Trichinellosis in China

**DOI:** 10.3389/fmicb.2017.01472

**Published:** 2017-08-02

**Authors:** Xue Bai, Xiaoxiang Hu, Xiaolei Liu, Bin Tang, Mingyuan Liu

**Affiliations:** ^1^Key Laboratory of Zoonosis Research, Ministry of Education, Institute of Zoonosis, Jilin University Changchun, China; ^2^Jiangsu Co-innovation Center for Prevention and Control of Important Animal Infectious Diseases and Zoonoses Yangzhou, China

**Keywords:** trichinellosis, diagnosis, vaccine, immune-related disease, China

## Abstract

Trichinellosis, caused by *Trichinella*, is an emerging or re-emerging zoonotic parasitic disease, which is distributed worldwide with major socio-economic importance in some developing countries. In particular, it has been calculated that more than 40 million people are at risk of *Trichinella* infection in China. This review summarizes the current information on the epidemiology, laboratory diagnosis and vaccines of trichinellosis in China. Moreover, study of the treatment potential of using *Trichinella* for immune-related diseases and cancer, as well as the transcription and post-transcription modification of *Trichinella* were also collected, providing viewpoints for future investigations. Current advances in research will help us to develop new strategies for the prevention and control of trichinellosis and may potentially yield biological agents for treating other diseases.

## Introduction

Trichinellosis is a worldwide food-borne parasitic disease caused by eating raw or undercooked meat containing the infective larvae of *Trichinella* nematodes (Rainova et al., 2016). Pork and its products are the main sources of infection (Sofronic-Milosavljevic et al., 2017). *Trichinella* has a wide range of hosts and can infect more than 150 species of animals, including humans. It is evaluated that around 11 million people may be infected by *Trichinella* (Dupouy-Camet, 2000). The International Commission on Trichinellosis (ICT) reported total about 65818 cases of human trichinellosis from 1986 to 2009 (Murrell and Pozio, 2011). In 2014, the Food and Agriculture Organization of the United Nations (FAO) and the World Health Organization (WHO) composed a list of 24 parasites ranked according to nine global criteria, *Trichinella spiralis* ranked the first in international trade (FAO/WHO, 2012).

At present, China is one of a few of countries with the highest number of cases of trichinellosis in the world. According to notice No.1149 announced by the Ministry of Agriculture in 2009, trichinellosis was included in the “list containing 26 kinds of the most hazardous zoonoses”. Trichinellosis also have important influence on animal production, food safety and trade in China (Dorny et al., 2009). The cost of prevention and control of *Trichinella* remains high. According to preliminary statistics, China spends 2.2 billion CNY on the inspection and control of *Trichinella* per year (Jen and Chen, 2017). Therefore, controlling trichinellosis is of great significance to the meat industry and human health. In this review, we systematically introduce the recent progress in trichinellosis research.

## Epidemiology

Nematodes of the genus *Trichinella* are one of the most worldwide zoonotic pathogens (Knopp et al., 2012). Today, nine species and three genotypes are recognized in this genus (Pozio and Zarlenga, 2013; Korhonen et al., 2016). At present, out of the 16 isolates obtained from mainland China, 13 have been identified as *T. spiralis*, and these specimens were collected exclusively from pigs from all over the country, including six provinces (Heilongjiang, Liaoning, Henan, Shaanxi, Hubei, and Yunnan) and a municipality (Tianjin). The remaining two isolates from dogs and one from cat were identified as *T. nativa*, and were collected from two provinces (Heilongjiang and Jilin) in northeast of China (Takahashi et al., 2000). Aside from *T. spiralis* and *T. nativa*, *T. pseudospiralis*, and *T. papuae* infections have also been reported in Chinese Taiwan as a result of ingesting raw soft-shelled turtles (Lo et al., 2009). To date, *Trichinella* has been found in 15 species of animals, such as pig, dog, cat, rat, cow, fox, and bear et al., which are distributed throughout China, except in the Hainan province (Liu and Boireau, 2002).

**FIGURE 1:**
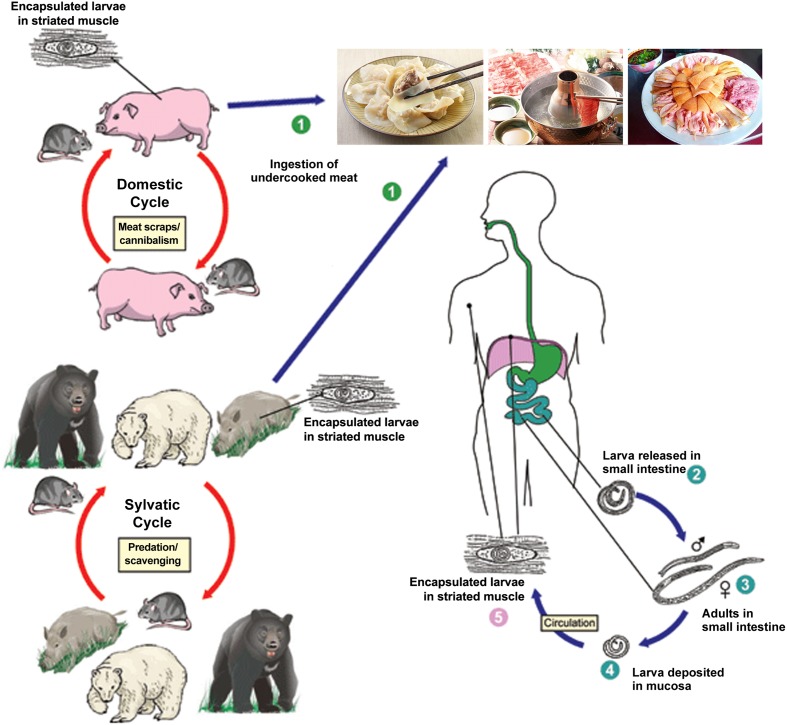
Schematic representation of the main sources of infection for trichinellosis and life cycle of *Trichinella spiralis* in host (cite by https://www.cdc.gov/parasites/trichinellosis/biology.html). ➀ Humans are infected by ingesting encapsulated larvae of *Trichinella* when eating improperly processed meat; ➁ After exposure to gastric acid and pepsin, the larvae are released from the capsule and invade the small bowel mucosa; ➂ Larvae develop into adult worms and copulate in the small intestine; ➃ Larvae are released and migrate into the vessels and lymphatics; ➄ Larvae reach and settle in the striated muscles where they are encapsulated.

From 2001 to 2004, the Ministry of Health surveyed the prevalence of parasitic diseases across China. The survey revealed an increasing occurrence of foodborne parasitic diseases where trichinellosis is ranked as one of the top three, with an increase of 69.44% and an estimated increase in infections of approximately 20 million people compared to the first national survey (CDC, 2005). During 1964–2011, more than 600 outbreaks of human trichinellosis were documented in mainland of China, affecting 38,797 people and causing 336 deaths (Wang and Cui, 2001; Wang et al., 2006; Cui et al., 2011; Zheng et al., 2011). In recent years, trichinellosis outbreaks have mainly occurred in Yunnan Province, such as the outbreaks in Lanping and Lancang County that occurred in 2009 and 2013, respectively (Jen and Chen, 2017). The high prevalence of trichinellosis in China is related to pig breeding and eating habits (**Figure 1**). For example, some inhabitants consume wild animals, raw meat and under-cooked foods such as dumplings or scalded dog meat as delicacies, however, there has not been mandatory test for *Trichinella* larvae in meats except pork in China at present (Wang et al., 2007; Li et al., 2010).

## Diagnosis

In 2016, the World Organization for Animal Health (OIE) reported that digestion method is the best testing method for diagnosing trichinellosis (OIE, 2016). This method involves routine examination of *Trichinella* larvae in muscle tissues for either food safety or disease surveillance and shows good sensitivity and effectiveness in preventing clinical trichinellosis (OIE, 2016). Currently, there are three national standards for the detection of trichinellosis in China: Diagnostic techniques for *T. spiralis* in swine (GB/T 18642-2002) by the General Administration of Quality Supervision, Inspection and Quarantine; Diagnosis of trichinellosis (WS 369-2012) by the Ministry of Health; and Technical standard for management of trichinellosis outbreak (WS 470-2015) by the National Health and Family Planning Commission (Xu et al., 2002; Wang et al., 2012, 2015). Otherwise, in order to better detect *Trichinella*, serology and molecularbiologic methods have been developed.

### Immunoassays

Animals can be tested for the presence of antibodies against *Trichinella* in serum or meat juice in antemortem or postmortem examinations (Nöckler et al., 2005). A variety of immunological assays have been developed for the detection of *Trichinella* infection in domestic and wild animals. Among these tests, ELISA is the most common method for detecting *Trichinella* infection, and ELISA based on excretory/secretory antigens from ML is the only immunological assay endorsed for surveillance and epidemiological investigations of infections and outbreaks in domestic animals and wildlife by ICT (Gottstein et al., 2009). The disadvantage of using ML-ES ELISA is the high rate of false negative results when animals are in the early stage of infection (Gamble et al., 2004; Yang et al., 2016b). ELISA based on adult worm (AW) ES antigens showed a promising potential for the early and specific serodiagnosis of trichinellosis (Sun et al., 2015b). In addition, a sandwich ELISA based on IgY polyclonal antibodies and IgM monoclonal antibodies was established to detect CAg (Liu L.N. et al., 2013). This method was successfully employed for early detection of *T. spirali*s in mice and may provide an alternative and more reliable assay.

To improve the ES-ELISA, cDNA libraries of different developmental stages of *Trichinella* were screened using the serum of pigs at different days post-infection (dpi), and some immunodominant antigens of *T. spiralis* were evaluated to detect *Trichinella* infection, showing a promising diagnostic potential (Zhu et al., 2005; Liu et al., 2007; Wu et al., 2009; Liu P. et al., 2013). Interestingly, ELISA based on antigenic molecules (T668, *Ts*-CLP and 31 kDa antigens) could detect *Trichinella* infection earlier than ES antigens (Cui et al., 2015; Tang et al., 2015). In addition to these efforts, identification of immunodominant linear epitopes on antigen by monoclonal antibodies and sera from different host infected *Trichinella* will also greatly improve detection of *T. spiralis* using ELISA (Yang et al., 2016c).

ES proteins released by *Trichinella* induce a strong and specific humoral immune, and molecules containing ES are ideal as diagnostic antigens. Two-dimensional electrophoresis (2D) combined with western blot and mass spectrometry was used to screen the early diagnostic antigen from ML ES, identifying five proteins (Wang L. et al., 2013, 2014). Furthermore, several proteins (deoxyribonuclease II and serine protease family protein, et al.) were identified from the intestinal infective larvae (IIL) and adult worms as ES antigens, and these may also serve as potential early diagnostic antigens for trichinellosis (Sun et al., 2015a; Liu et al., 2016; Wang Z.Q. et al., 2017).

An emerging rapid and easy alternative to ELISA is immunochromatographic strips, which detect *Trichinella* antibody using colloidal gold labeling ES antigens. Zhang et al. prepared an immunochromatographic strip for rapid diagnosis and successfully detected serological trichinellosis in swine. The strips could serve as a substitute for diagnosis and surveillance of trichinellosis when lacking equipment (Zhang et al., 2006). Early diagnosis of trichinellosis is still facing serious challenges, identification of the antigens at different stages using molecular biology and immunology methods will provide a solid base for the further development of serological tools.

### DNA Methods

PCR-based methods are most commonly used in live animal slaughter for meat products. LAMP is a novel nucleic acid detection method that can be performed within 1 h under isothermal conditions (Notomi et al., 2000). The LAMP assay also was developed for detection of *T. spiralis* larvae infection, and showed high sensitivity with detecting *T. spiralis* in all mouse muscle samples infected with 10 larvae on 20 dpi, demonstrating a valuable means to directly detect larvae during meat inspection (Li et al., 2012). A duplex PCR based on liquid gene chip technique was also developed for detecting *T. spiralis* in foods using primers designed from the *T. spiralis* 18S rDNA gene sequences, and the detection limit of this method is 8-fold more sensitive than using agarose gel (Yang P. et al., 2010).

## Vaccines

Benzimidazole derivatives are principal anthelmintic drugs which are safe, cheap and effective for the treatment of human trichinellosis (Dupouy-Camet et al., 2002). Some new drug targets are being screened, e.g., Cathepsin F of *T. spiralis* is a major virulence factor shown to interact with more than ten kinds of drugs, indicating potential drug target for treatment. Although the control strategy of parasites primarily relies on drugs against a broad spectrum of parasites, the emergence of drug-resistant parasites has threatened their sustained use (Roberts, 2005; Schellenberg et al., 2006; Vercruysse et al., 2007). In this circumstance, the development of effective vaccines against *Trichinella* infection in livestock and humans is a promising strategy to control this parasite (Jacob et al., 2013). However, no effective vaccines are currently available to fully protect against *Trichinella* infections, except for some protective effects observed only in rat or pig models (Hotez et al., 2008).

### Recombinant Protein Vaccine

Researchers have used different antigens to construct recombinant protein vaccines, most of which show some protection against *Trichinella*. A recombinant vaccine using combined sequences of the *T. spiralis* serine protease (r*Ts*-Adsp) and Nudix hydrolase (*Ts*Nd) can limit the invasion of *T. spiralis* in mice (Feng et al., 2013; Long et al., 2014). The *T. spiralis* adult somatic protein *Ts*14-3-3 is an immunodominant protein identified by early infection sera, and immunization with *Ts*14-3-3 have shown promising results for preventing swine trichinellosis propagation (Yang et al., 2015, 2016a).

Although these vaccines appear promising, the immunoprotective effects still depends on the type of antigen, adjuvants and the delivery route used to trigger robust immune response (Mohsen et al., 2017). In addition to a variety of traditional adjuvants, new adjuvants consisting of cytokines, nanoadjuvants and toll-like receptor agonists have made great progress in experimental model (Qi and Fang, 2011). Compared to the Montanide ISA201 and Freund’s adjuvant formulated vaccines, the Montanide IMS 1313 NPR VG plus r*Ts*-serpin mixture showed higher humoural and cellular immunity as well as a protective immune response against *Trichinella* infection in mice (Xu et al., 2017b).

### DNA Vaccine

DNA vaccines can induce intense long-term immune responses and do not require booster immunization such as live vaccines. Additionally, DNA vaccines are usually well-tolerated by the animal and thereby safe for use with little risk. In addition, the DNA molecule itself can enhance the immune response as an adjuvant (Heppell and Davis, 2000).

DNA vaccines can contain some antigenic molecules, such as *Ts*Nd mentioned above and *Ts*-NBLsp (the serine protease of *T. spiralis* new-born larvae) (Liu et al., 2015a; Xu et al., 2017a). Vaccination of mice with pcDNA3.1-*Ts*Nd and *Ts*-NBLsp displayed 53.9 and 77.93% reductions in larval burden, respectively, which are higher protective levels than recombinant protein vaccine.

Attenuated *Salmonella typhimurium* is an effective carrier for oral delivery of heterologous antigens to induce the immune response. *S. typhimurium* has been investigated as a vaccine carrier for viruses, bacteria, gene therapy and parasites, inducing long-lasting systemic and mucosal humoral immune responses, and providing a rational design for efficient vaccine (Cazorla et al., 2015). DNA vaccines using *Ts*Pmy, *Ts*Nd, *Ts*87, and *Ts*-cystatin were made and delivered orally using attenuated live *Salmonella typhimurium* to provide partial protection against *T. spiralis* infection in mice, suggesting that this may be a promising approach for controlling trichinellosis in human and domestic animals (Yang Y. et al., 2010; Liu et al., 2014, 2015b; Wang et al., 2016).

## Immune-Related Diseases and Cancer

*Trichinella* infection or its derived antigens can induce various immunity-related diseases, including experimental colitis and airway allergic inflammation (Wang M. et al., 2017). One study demonstrated the intervening effect of *T. spiralis* infection in the mouse TNBS-IBD model (Zhao et al., 2013). In IBD therapy using *Trichinella* or ES products (ESP), negative regulation of TLR signaling is critical for reducing the expression of genes involved in inflammation and pro-inflammatory cytokine production (Sun et al., 2011). ESP induced macrophage towards the alternatively activated macrophage, suggesting that ES products have the ability to affect macrophages, thereby influencing the host’s immune response and therapeutic potential (Bai et al., 2012). ESP also exhibits anti-inflammatory properties in the septic mouse model, improving survival, reducing organ damage and enhancing bacterial clearance (Du et al., 2014; Chen et al., 2016; Li et al., 2016). In addition to inducing anti-inflammatory immune response, *Trichinella* and its ESP also have the ability to reduce immune rejection. Mice that were infected with *T. spiralis* showed higher survival rates after solid organ transplantations, suggesting that the ESP released by *T. spiralis* may provide an anti-allograft rejection immune response (Deng et al., 2016).

The immunoregulation effect of some immunomodulatory molecules has also been demonstrated, such as the recombinant 53-kDa protein of *T. spiralis* (r*Ts*-p53) in the TNBS-IBD and septic mouse models (Du et al., 2011; Chen et al., 2016). The effects of *T. spiralis* cathepsin B-like protein (r*Ts-*CPB) on intestinal ischaemia/reperfusion injury through altering macrophage phenotypes were also investigated, and the results showed that r*Ts-*CPB significantly relieve intestinal injury and protect intestinal function (Liu W.F. et al., 2015).

*Trichinella spiralis* infection can inhibit tumor growth by cytokines released by activated immune cell. In addition, molecules from *T. spiralis* can induce tumor or cancer cell apoptosis by inducing apoptosis-related genes, mitochondrial pathways or the death receptor pathway (Wang et al., 2009). In a screen for anti-tumor genes using a T7 phage display cDNA library with organic phase multi-cells, the protein named A200711 showed the potential to induce H7402 cells apoptosis (Duan et al., 2013; Wang X.L. et al., 2013). These studies suggest that *T. spiralis* should be considered as a potential source of an anti-tumor protein that may have therapeutic applications.

## Transcription Small RNA and Post Transcription Modification

Currently, there are stage-specific gene expression results using various immunological and cDNA cloning method; however, genome-wide transcriptome and expression patterns of *T. spiralis* remain largely unknown. Based on the draft genome of *T. spiralis*, the global gene expression profile in the three different developmental stages of *T. spiralis* was analyzed using digital gene expression (DGE) analysis in our group. The transcriptomic analysis of *T. spiralis* revealed that many genes related to metabolic and biological pathways in the genome were developmentally regulated (Liu et al., 2012). Small non-coding RNAs (sncRNAs) are involved in gene silencing through transcriptional destabilization or translational repression (Mokhtarzadeh et al., 2017). In our previous study, we identified 21 conserved miRNAs related to 13 previously identified metazoan miRNA families as well as 213 miRNAs unique to *T. spiralis* in three developmental stages, with some miRNAs showing clear stage-specific expression patterns (Liu et al., 2011). These data provide a basis for further understanding molecular mechanisms of parasite biology and functional evolution of miRNAs in parasitic nematodes.

DNA methylation plays a crucial role in modulating gene expression under various conditions, and is suggested to be related with transitions between life cycle stages in parasitic nematodes (Hewezi et al., 2017). Gao et al. (2012) presented the first study to confirm the existence of DNA methylation in *T. spiralis* using MethylC-seq, and they observed a drastic increase in DNA methylation during the transition from the new-born to mature stage and found parasitism-related genes that show changes in DNA methylation status between life cycle stages. Based on these results, authors suggested that interference DNA methylation processes may be a beneficial strategy in developing therapeutics to control parasite infection.

## Conclusion

Although some trichinellosis control programs have been implemented and advances have been made to better understand *T. spiralis* at the molecular level, trichinellosis remains prevalent in China due to the absence of systematic interventions. The wide distribution of *Trichinella*, dietary habits, the lack of meat safety regulation, and without developed techniques for detection and treatment are contributing to the prevalence of trichinellosis. Importantly, new strategies of combining non-polluted domestic animal breeding with the use of vaccines may represent a viable alternative to block the transmission of *Trichinella* and ensure meat safety. By the end of 2015, the OIE set up a total of 12 reference laboratories and 3 collaborating centers in China. Among them, a center for foodborne parasites in the Asian-Pacific region center was set up in Jilin University to provide comprehensive monitoring and detection of foodborne parasitic diseases, including trichinellosis. New methods for effective diagnosis and prevention of trichinellosis are being developed in cooperation with domestic and international research institutions.

Moreover, the rapid development in *Trichinella-*omics research has provided a new opinion for understanding the biology of *Trichinella* and screening target molecules to develop new anti-parasitic agents. In addition, identified *Trichinella* molecules also serve as protective agents for immune-related disease and cancer in humans.

## Author Contributions

XB and XH wrote the initial draft of the paper. XL organized and proofread the paper. BT helped to draft the figure. ML approved the version to be published. All authors read and approved the final manuscript.

## Conflict of Interest Statement

The authors declare that the research was conducted in the absence of any commercial or financial relationships that could be construed as a potential conflict of interest.
